# Radix Scutellariae Ameliorates Stress-Induced Depressive-Like Behaviors via Protecting Neurons through the TGF*β*3-Smad2/3-Nedd9 Signaling Pathway

**DOI:** 10.1155/2020/8886715

**Published:** 2020-11-13

**Authors:** Fan Zhao, Chenyiyu Zhang, Dong Xiao, Weihua Zhang, Liping Zhou, Simeng Gu, Rong Qu

**Affiliations:** ^1^College of Chinese Medicine, College of Integrated Chinese and Western Medicine, Nanjing University of Chinese Medicine, Nanjing 210046, China; ^2^Jiangsu Collaborative Innovation Center of Chinese Medicinal Resources Industrialization, National and Local Collaborative Engineering Center of Chinese Medicinal Resources Industrialization and Formulae Innovative Medicine, Nanjing University of Chinese Medicine, Nanjing 210046, China; ^3^Department of Psychology, Jiangsu University Medical School, Zhenjiang 210023, China

## Abstract

Chronic stress can impair hippocampal neurogenesis, increase neuronal apoptosis, and cause depressive-like behaviors. Our previous studies found that Radix Scutellariae (RS) can rescue the stress-induced neuronal injury, but the mechanism is not clear. Here, we continued to investigate the underlying antidepressant mechanisms of the RS extract. A 7-week chronic unpredictable mild stress (CUMS) procedure was used to establish a murine depression model. 0.75 g/kg or 1.5 g/kg RS was administered daily to the mice during the last 4 weeks. Depressive-like behaviors were evaluated by the sucrose preference test (SPT), forced swimming test (FST), open field test (OFT), and tail suspension test (TST). The neuroprotective effect of RS was evaluated with the expression of hippocampal neuron-related markers and apoptosis-associated proteins by Nissl staining, immunohistochemistry, and western blot. Transforming growth factor-*β*3 (TGF*β*3) pathway-related proteins were detected by western blot. Results showed that RS could ameliorate depressive-like behaviors, increase the expression of the antiapoptotic protein B-cell lymphoma 2 (BCL-2), reduce the expression of the proapoptotic protein BCL-2-associated X (BAX), and increase the number of doublecortin- (DCX-), microtubule-associated protein 2- (MAP2-), and neuronal nucleus- (NeuN-) positive cells in the hippocampus. Moreover, RS could reverse the CUMS-induced decrease of TGF*β*3 protein, promote the phosphorylation of SMAD2/3, and increase the expression of downstream NEDD9 protein. These results suggest that RS could exert antidepressant effects via protecting neurons. And the molecular mechanism might be related to the regulation of the TGF*β*3-SMAD2/3-NEDD9 pathway.

## 1. Introduction

Major depressive disorder (MDD) is an affective disorder with a high risk of morbidity and mortality. Depression produces the greatest decrement in health compared with the chronic diseases angina, arthritis, asthma, and diabetes [1], making it one of the most prevalent health-related causes of human suffering [2]. However, the mechanism of depression are far from clear, the most widely accepted theory about the mechanism of depression points to the monoamine neurotransmitters, and the first line of treatment of depression is the monoamine reuptake inhibitors [3]. However, even though there are many achievements in pharmacological and psychological therapies, an estimated 44% of patients do not respond to two consecutive antidepressant therapies and an estimated 33% do not respond to four consecutive antidepressant therapies [4]. Therefore, it is necessary to look for safe and effective drugs to treat MDD.

**Figure 1:**
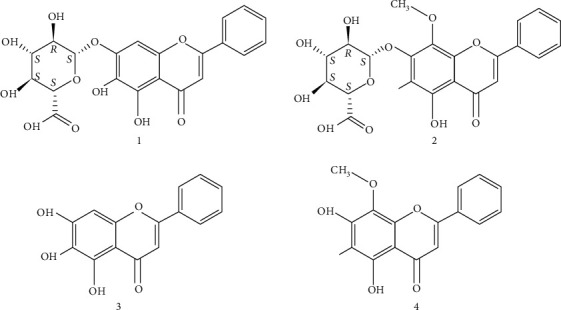
Chemical structures of the 4 identified compounds in RS: baicalin (1), wogonoside (2), baicalein (3), and wogonin (4).

Radix Scutellariae (RS), one of the components of Xiao-chai-hu-tang (XCHT), is a dry root of the Lamiaceae plants, *Scutellaria baicalensis* Georgi. XCHT is a famous Chinese herbal formula that has been widely used clinically in depressive disorders in China. In addition, XCHT has been shown to significantly ameliorate depressive-like behavior in several animal models of depression by altering the serotoninergic system and neurotrophic factors in the hippocampus [5, 6]. Zhang and collaborators used an orthogonal array design experiment to show that RS, ginseng, and Radix Glycyrrhizae are supposed to be the core in compatibility of XCHT in antidepressant therapy [7]. Furthermore, baicalin (Figure 1), the major polyphenol component of RS, has potent antidepressant effects by upregulating the expression of *α*-amino-3-hydroxy-5-methyl-4-isoxazolepropionic acid (AMPA) receptors and the suppression of neuronal apoptosis in CUMS-treated rats [8]. Moreover, baicalin can facilitate the differentiation of neural stem/progenitor cells to neurons and stimulate hippocampal neurogenesis in adult rats [9]. These studies suggest that RS has a well-founded antidepressant effect and the therapeutic effect of RS on depression is associated with the regulation of neurogenesis and apoptosis. However, the details underlying the molecular mechanisms are still elusive.

Recent studies have shown that neurogenesis theory is suggested to compensate for the limitations of the monoamine theory in depression [10]. In the adult hippocampus, neurogenesis is functionally related to regulation of the hypothalamic-pituitary-adrenal (HPA) axis, inflammatory processes, cognitive functions, and other aspects that contribute to etiological factors that lead to MDD and promote recovery from MDD [11]. The human transforming growth factor-*β* (TGF*β*) signaling pathway might regulate the proliferation of neuroepithelial stem cells, which leads to enhanced neurogenesis [12]. Using biotin label-based antibody protein chips to detect the expression levels of TGF*β*3 in hippocampal tissues, it has been shown that the expression levels of TGF*β*3 were decreased in the CUMS model; however, electroacupuncture therapy can improve the depressive-like state via promoting neurogenesis which might be associated with its effect on upregulating TGF*β*3 protein level [13]. In addition, TGF*β*s were shown to inhibit apoptosis, which contribute to their neuroprotective effects [14]. Importantly, it has been reported that RS can regulate the TGF*β* signaling pathway [15]. However, the relationship between the regulation of RS on the TGF*β* signaling pathway and the antidepressant effect of RS associated with neuroprotection on neurons has not been reported in the literature.

So we assessed the antidepressant effects of the RS extract on behaviors in a CUMS mouse model and explored the underlying mechanism associated with the TGF*β* signaling pathway. Furthermore, we used the HPLC fingerprint to detect the main components of RS.

## 2. Materials and Methods

### 2.1. Radix Scutellariae Extract and Chemicals

RS was supplied by Nanjing University of Chinese Medicine and prepared as previously described [16]. Extracts of RS were prepared by macerating the dried herb in distilled water for 2 h and then boiling two times (100 g/800 ml for 2 h; 100 g/800 ml for 1 h). The two decoctions were mixed and filtered, then concentrated to water extracts (0.15 g/ml), and stored in a refrigerator. Baicalin, wogonoside, baicalein, and wogonin were purchased from Liangwei Biological Technology Co., Ltd. (Nanjing, China). The purity of each compound was >98%, determined by HPLC analysis. The chemical structures of these reference compounds are shown in Figure 1.

### 2.2. Animals

Fifty adult male ICR mice, aged 6-7 weeks and weighing 18-22 g, were purchased from the Jiangsu Provincial Experimental Animal Center (Nanjing, China). They were adapted to animal facilities for 1 week before the experiment. The animals were placed under a 12/12 h light/dark cycle (7 am/7 pm) and the prescribed temperature conditions (22 ± 2°C). Food and water were provided free of charge. All animal experiments are conducted in accordance with the National Institutes of Health guidelines (NIH publication no. 80-23, 1996 revision) and in accordance with the PRC laboratory animal care and use regulations.

### 2.3. Chronic Unpredictable Mild Stress Procedure

**Figure 2:**
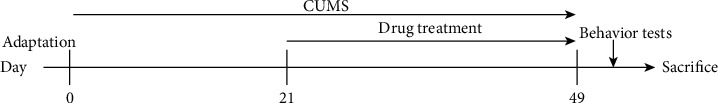
The schematic representation of the experimental procedure.

The CUMS program is a slight improvement over the published program described by Willner and our previous research [17, 18]. This paradigm is designed to maximize unpredictability, as the application of stressors seems to be random and at different times. The CUMS mice were exposed to stress twice a day, and the CUMS program was applied for seven weeks (Figure 2). All procedures are conducted in isolated rooms adjacent to the house, with minimal animal handling or transportation requirements.

### 2.4. Drug Administration and Treatment

After the depressive-like behavior was observed in the third week of the CUMS paradigm, therapeutic administration was given daily for 4 weeks. Fluoxetine hydrochloride (Flu) (positive control drug) is produced by Changzhou Siyao Pharmaceutical Co., Ltd. (Changzhou, P.R. China). Flu is dissolved in normal saline. All drugs and the vehicle (0.9% normal saline) were administered in a volume of 10 ml/kg of body mass via intragastric administration between 8:00 am and 10:00 am. The control group (*n* = 10/group) and CUMS group mice received only normal saline, and the CUMS+Flu group (20 mg/kg) and CUMS+RS group (0.75 g/kg and 1.5 g/kg) mice received the related drugs. Behavioral tests were performed 1 hour after the last administration of d49.

### 2.5. Behavioral Tests

Behavioral tests mainly include the sucrose preference test (SPT), OPT, FST, and TST. The details of the test methods were performed as in the previously published articles [18, 19, 20]. Briefly, the mice were trained to adapt to the sucrose solution for 24 h; then, the SPT lasts for another 24 h. The OPT was carried out in a 40 × 60 × 50cm black metal shell. The mice were free to explore for 6 minutes, and the number of crossings was recorded for the last 4 minutes. The FST was carried out in a transparent plexiglass container (20cmhigh × 14cmdiameter). Each mouse was individually subjected to swimming freely for 6 minutes, and the immobility time was recorded for the last 4 minutes. In the TST, the tail of the mice was fixed 15 cm away from the table for 6 minutes, and the immobility time of the last 4 minutes was recorded.

### 2.6. Western Blotting

The western blotting method was the same as in our previous publications [18]. On day 50, mice were sacrificed after being deeply anesthetized with sodium pentobarbital (50 mg/kg, i.p.). Brain tissue and hippocampal tissue were rapidly dissected on ice and homogenized. Western blot analysis was performed on three hippocampal tissues from each group. The hippocampal tissue was homogenized with calving tissue buffer (*w* : *v* 1 : 5), and the protein concentration was determined according to the manufacturer's instructions using the BCA protein detection kit (Beyotime, Haimen, China). The proteins were run on the SDS-PAGE gel and transferred to a balanced polyvinylidene fluoride (PVDF) membrane (Millipore, Billerica, MA, USA). The main antibodies were anti-HEF1 (ab18056), anti-TGF*β*3 (ab15537), anti-Smad2/3 (ab202445), anti-p-Smad2/3 (ab63399), anti-BCL-2 (ab196495), and anti-BAX (sc-20067). After washing, the membrane was incubated with an HRP-conjugated secondary antibody at room temperature for 2 h and developed with an enhanced chemiluminescence (ECL) kit (Millipore, Billerica, MA, USA). ImageJ software was used to analyze the intensity of the blots.

### 2.7. Immunohistochemistry

Immunohistochemistry was performed as previously reported [21]. The brain was fixed with 10% formalin for 24 hours and embedded in paraffin. Then, the tissue sections were placed in a rotary slicer for immunohistochemical procedures. The brain tissue was cut into 4.5 *μ*m, and the sections containing the hippocampus were incubated with primary antibodies: anti-DCX (ab18723), anti-MAP2 (ab32454), and anti-NeuN (ab177487). After washing with PBS, the sections were incubated with the appropriate secondary antibody. Finally, sections were displayed using 3,3′-diaminobenzidine solution (DAB). The positive expression of target proteins in the dentate gyrus (DG) and cornu ammonis (CA) 1 in the hippocampus was observed under a 10x light microscope. Brown DAB staining was considered positive staining. Then, ImageJ software was used to automatically quantify NeuN-, DCX-, and MAP2-positive cells, and the density of these three positive cells in DG was calculated in three brain sections of each group.

### 2.8. Nissl Staining

Brain tissues were cut into 4.5 *μ*m in the coronal plane for Nissl staining [22]. The morphological changes in the CA1 region and CA3 region of the hippocampus were observed with a light microscope. The number of positive cells was calculated in three brain sections of each group.

### 2.9. HPLC Analysis and Method Validation

Chromatographic analysis was performed on a Waters 2695 Alliance HPLC system (Waters Corp., Milford, MA, USA) equipped with an Apollo C18 column (250mm × 4.6mm, 5 *μ*m). The mobile phase consisted of acetonitrile (A) and 0.1% aqueous formic acid (B). The gradient elution program was as follows: 0-5 min (95% B), 5-50 min (95-50% B), and 50-60 min (50–0% B). The column temperature, flow rate, injection volume, and detection wavelength were set at 30°C, 1.0 ml/min, 10 *μ*l, and 274 nm, respectively. Validation was performed to verify the HPLC methods with pretreatment methods by evaluating the linearity, LOD, LOQ, accuracy, precision, stability, and recovery. Linear calibration curves were established by plotting the peak area (*Y*) versus the corresponding concentration (*X*, *μ*g/ml) of baicalin, wogonoside, baicalein, and wogonin, respectively. The correlation coefficients in calibration curves were estimated to examine the linearity. LOD and LOQ at the lowest concentration of calibration standards were calculated with the slope and standard deviation of the analytical response (LOD = 3*σ*/S, LOQ = 10*σ*/S) [23]. Precision was estimated from the data of intraday and interday tests once a day for three days. After the addition of samples, the stability was observed at 0, 2, 4, 8, 12, 24, and 48 h. Repeatability was confirmed by analyzing six independently prepared solutions of sample RS. The relative standard deviation (RSD%) of the peak area for each marker compound was taken as a measure.

### 2.10. Statistical Analysis

GraphPad Prism 6.0 software was used for the analysis. Statistical analyses were performed via one-way ANOVA followed by Tukey's test. The significance threshold used was *p* < 0.05. All data were reported as mean ± SEM.

## 3. Results

### 3.1. HPLC Analysis of RS and Method Validation

**Figure 3:**
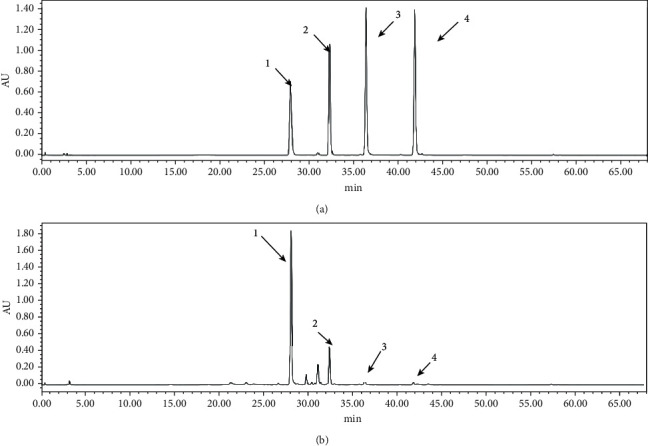
HPLC chromatograms of solution of (a) standards and (b) samples at 274 nm. Peaks: baicalin (1), wogonoside (2), baicalein (3), and wogonin (4).

A representative chromatogram of the RS extract along with the chromatogram for the standard compounds is presented in Figure 3. The average proportions of the flavonoids in the extracts of baicalin, wogonoside, baicalein, and wogonin were 217.77 ± 1.96%, 42.77 ± 0.38%, 7.14 ± 0.10%, and 2.08 ± 0.02% (*w*/*w*), respectively (Table 1), which is consistent with the results of previous studies [24, 25]. The HPLC method was validated by evaluating the linearity, limit of detection (LOD), limit of quantitation (LOQ), precision (interday and intraday), repeatability, stability, and accuracy. All calibration curves exhibited good linearity with a correlation coefficient (*R*^2^) of greater than 0.999 (Table 2). The relative standard deviation (RSD) of the peak area for each marker compound was taken as a measure (Table 3). In intraday and interday tests, the RSD values of standards were in ranges of 0.65%-2.91% and 1.12%-1.69%, respectively. The stability was between 1.45% and 2.50%. In addition, the range of repeatability was 1.73%–3.22%.

### 3.2. Effects of RS on Body Weight

**Figure 4:**
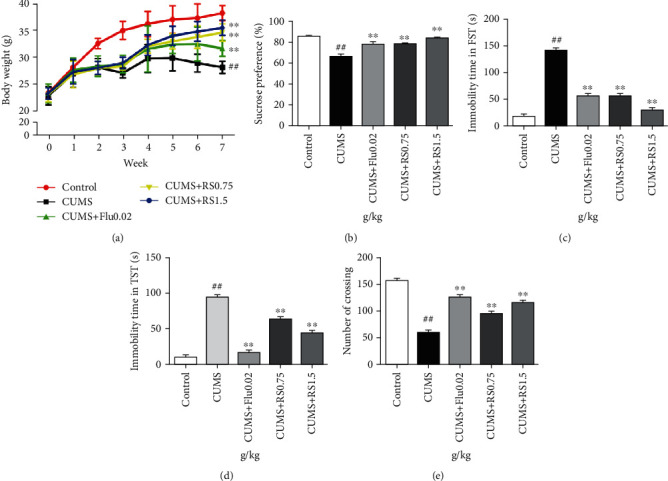
Effects of RS on body weight and behavioral studies: (a) effect of RS on body weight; (b) effect of RS on SPT; (c) effect of RS on immobility time in FST; (d) effect of RS on immobility time in TST; (e) effect of RS on the number of crossing in OFT. Results were reported as means ± SEM (*n* = 10). ^##^*p* < 0.01 versus the control group; ^∗∗^*p* < 0.01 versus the CUMS group.

The results of body weight are illustrated in Figure 4(a). Weight loss is a core feature of depression in the CUMS animal model [17]. There was no significant difference in the initial body weight of each group (*p* > 0.05). After three weeks of CUMS, the mice in the CUMS group gained less weight than the control group (*F*(4, 45) = 60.34, *p* < 0.01), and this condition continued for the following weeks. Four-week treatment of RS (0.75 and 1.5 g/kg) significantly attenuated the body weight reduction induced by CUMS (*F*(4, 45) = 73.00, *p* < 0.01).

### 3.3. Effects of RS on Depressive-Like Behaviors

As illustrated in Figures 4(b)–4(e), RS significantly ameliorated depressive-like behaviors induced by CUMS. Anhedonia, a prominent feature of depression in humans and rodents, manifested in rodents as a reduced preference for sucrose solutions over water [26]. After the 7-week CUMS program, the sucrose preference ratio (SPR) of CUMS-induced mice was lower (Figure 4(b)) (*F*(4, 45) = 21.29, *p* < 0.01) in comparison with that of the control group. However, in the administration of either 0.75 g/kg or 1.5 g/kg RS for 4 weeks, the SPR was higher than that of the CUMS group (*p* < 0.01).

We next used the FST and the TST, which are widely used for evaluating antidepressant activity, to test the effect of RS treatment on depressive-like behaviors in the CUMS model. Compared with the control group, immobility time in CUMS-induced mice was significantly increased (Figure 4(c)) (*F*(4, 45) = 573.0, *p* < 0.01) in the FST, which is consistent with the depressive phenotype. Compared with the CUMS-induced group, the 0.75 g/kg and 1.5 g/kg RS-treated groups had significantly reduced total immobility times (*p* < 0.01). As shown in Figure 4(d), the results of the TST were similar to the results of the FST. The CUMS-induced mice displayed a depressive-like phenotype as demonstrated by long immobility times in comparison with the control group (*F*(4, 45) = 408.7, *p* < 0.01). This depressive-like phenotype and long immobility times during the TST were significantly improved by the treatment with 0.75 g/kg or 1.5 g/kg RS (*p* < 0.01).

Depression is often accompanied by a decrease in spontaneous activity. As shown in Figure 4(e), the CUMS group showed a significant decrease (*F*(4, 45) = 232.6, *p* < 0.01) in spontaneous locomotor activity in the OFT when compared with the control group. Compared with the untreated and CUMS-induced group, both of the RS-treated (0.75 g/kg and 1.5 g/kg) groups had significantly increased spontaneous locomotor activity (*p* < 0.01). In summary, RS treatment can significantly improve the depressive-like behavior induced by CUMS in mice.

### 3.4. RS Rescued Neurons from CUMS-Induced Neuronal Injury and Apoptosis

**Figure 5:**
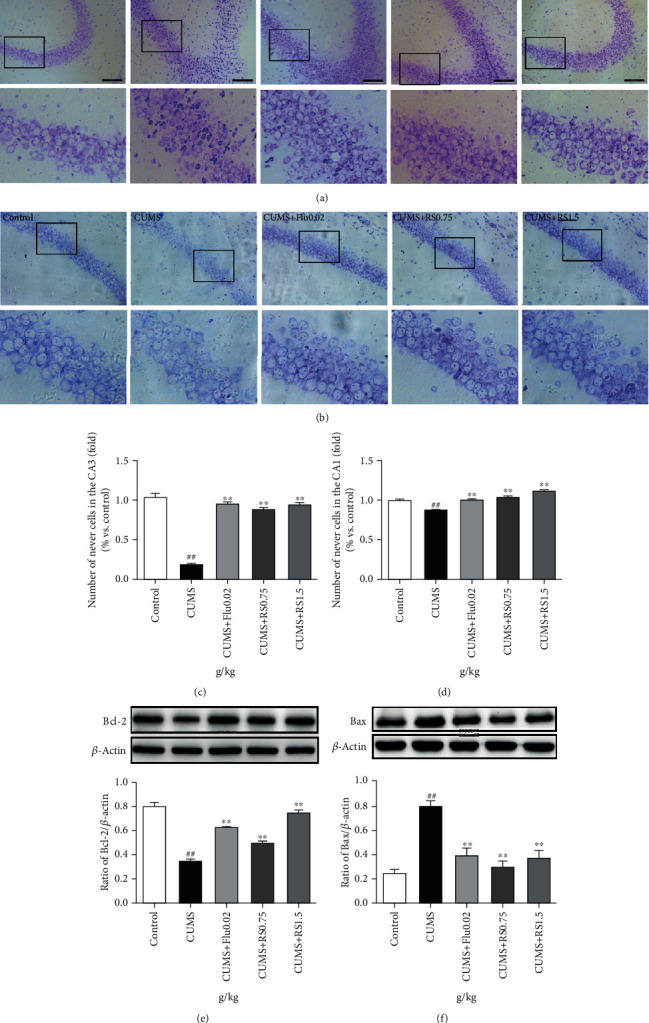
Nissl staining and effects of RS on apoptosis in the hippocampus. (a) Nissl staining in the CA3 area. (b) Nissl staining in the CA1 area. (c) Number of positive cells in the CA3 area. (d) Number of positive cells in the CA1 area. (e) Effect of RS on the Bcl-2 protein. (f) Effect of RS on the BAX protein. Results are reported as means ± SEM (*n* = 3). ^##^*p* < 0.01 versus the control group; ^∗∗^*p* < 0.01 versus the CUMS group. Scale bar, 100 *μ*m.

Nissl staining was used to evaluate the effect of RS treatment on CUMS-induced hippocampal neuronal injury. Nissl staining indicated that the CA3 (Figures 5(a) and 5(c)) and CA1 (Figures 5(b) and 5(d)) hippocampal areas of CUMS-induced mice had Nissl body loss, neuronal atrophy, and nuclear atrophy (CA3 region, *F*(4, 10) = 432.3, *p* <0.01; CA1 region, *F*(4, 10) = 70.96, *p* < 0.01). Compared with the CUMS-induced group, both of the RS-treated (0.75 g/kg and 1.5 g/kg) groups had increased numbers of normal Nissl bodies (*p* < 0.01).

To investigate the potential mechanism of RS treatment in alleviating CUMS-induced neuronal injury, western blot was used to detect the expression of BCL-2 and BAX in hippocampal tissue. BAX and BCL-2 are two opposite factors that affect whether cells enter the apoptotic process [27]. As shown in Figures 5(e) and 5(f), the BCL-2 levels of CUMS-induced mice decreased (*F*(4, 10) = 116.7, *p* < 0.01) and the BAX levels increased (*F*(4, 10) = 46.88, *p* < 0.01) compared with those of the control group. However, BCL-2 levels increased and BAX levels decreased (*p* < 0.01) in both of the RS-treated (0.75 g/kg and 1.5 g/kg) groups.

### 3.5. RS Protected Neurons via Promoting the Migration, Differentiation, and Maturation of Neural Stem Cells (NSCs) into Neurons

**Figure 6:**
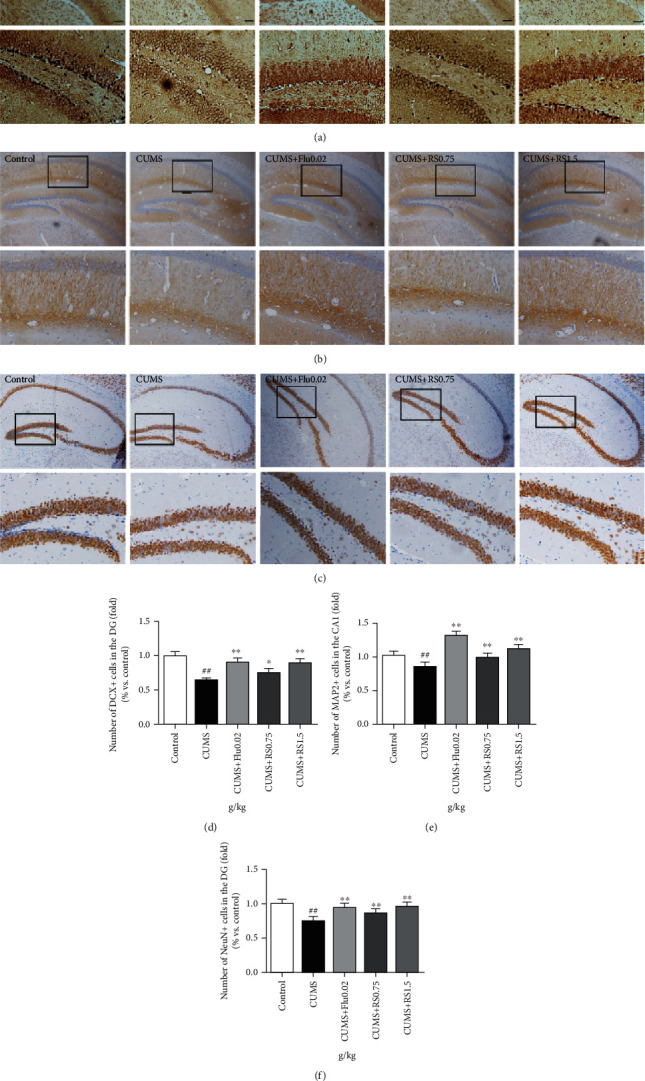
Effects of RS on DCX-, MAP2-, and NeuN-positive neuron numbers in the hippocampus. (a) Immunohistochemistry images of DCX-positive neurons in DG. (b) Immunohistochemistry images of MAP2-positive neurons in CA1. (c) Immunohistochemistry images of NeuN-positive neurons in DG. (d) Effects of RS on DCX-positive neuron numbers. (e) Effects of RS on MAP2-positive neuron numbers. (f) Effects of RS on NeuN-positive neuron numbers. Results are reported as means ± SEM (*n* = 3). ^##^*p* < 0.01 versus the control group, ^∗∗^*p* < 0.01 versus the CUMS group, and ^∗^*p* < 0.05 versus the CUMS group. Scale bar, 100 *μ*m.

Neurons are produced by proliferation, migration, and differentiation of NSCs, which occur in the subgranular zone (SGZ) of the DG in the hippocampus. To evaluate the effect of RS treatment on migration, differentiation, and maturation of NSCs to neurons, we detected the levels of DCX-, MAP2-, and NeuN-positive cells in the hippocampus, which are neuronal markers. Immunohistochemical analysis demonstrated a decreased number of DCX-positive cells (Figures 6(a) and 6(d)) (*F*(4, 10) = 39.54, *p* < 0.01) and NeuN-positive cells (Figures 6(c) and 6(f)) (*F*(4, 10) = 29.10, *p* < 0.01) in the DG region as well as a decreased number of MAP2-positive cells (Figures 6(b) and 6(e)) (*F*(4, 10) = 194.5, *p* < 0.01) in the CA1 region of CUMS-induced mice. With RS treatment for 4 weeks, the number of DCX-positive cells (0.75 g/kg RS, *p* < 0.05; 1.5 g/kg RS, *p* < 0.01), MAP2-positive cells (*p* < 0.01), and NeuN-positive cells (*p* < 0.01) significantly increased. These results suggest that CUMS reduces the number of hippocampal neurons by inhibiting the development and maturation of neurons and that the chronic treatment of RS promotes the migration, differentiation, and maturation of NSCs to neurons in CUMS-induced mice.

### 3.6. RS Exerted Neuroprotective Effect via Mediating the TGF*β*3-Smad2/3-Nedd9 Signal Pathway

**Figure 7:**
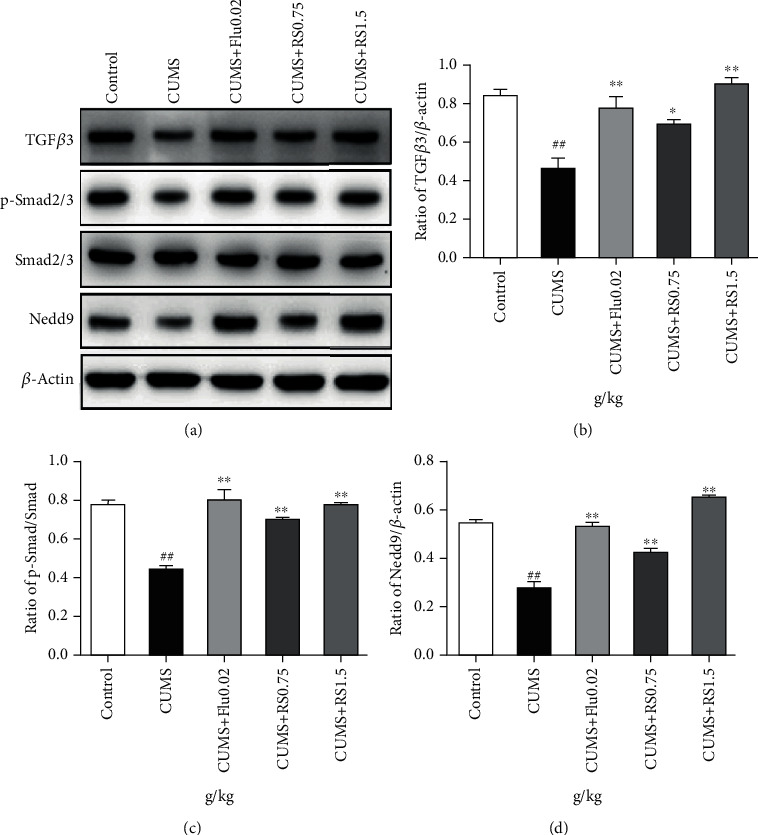
Effects of RS on the TGF*β*3-Smad2/3-Nedd9 signal pathway. (a) Effects of RS on the TGF*β*3-Smad2/3-Nedd9 signal pathway. (b) Effects of RS on the TGF*β*3 protein. (c) Effects of RS on the p-Smad2/3 protein. (d) Effects of RS on the Nedd9 protein. Results are reported as means ± SEM (*n* = 3). ^##^*p* < 0.01 versus the control group, ^∗∗^*p* < 0.01 versus the CUMS group, and ^∗^*p* < 0.05 versus the CUMS group.

The results of western blot showed that the CUMS procedure decreased the expression of TGF*β*3 (Figure 7(b)) (*F*(4, 10) = 15.04, *p* < 0.01), repressed the phosphorylation of SMAD2/3 (Figure 7(c)) (*F*(4, 10) = 27.38, *p* < 0.01), and reduced the levels of NEDD9 (Figure 7(d)) (*F*(4, 10) = 105.2, *p* < 0.01) compared with the control group. However, these changes in expression of proteins of the TGF*β*3-SMAD2/3-NEDD9 signaling pathway were restored in the RS groups. The expression of TGF*β*3 (0.75 g/kg RS, *p* < 0.05; 1.5 g/kg RS, *p* < 0.01), SMAD2/3 (*p* < 0.01), and NEDD9 (*p* < 0.01) increased. These results suggest that the neuroprotective effect of RS may be associated with the modulation of the TGF*β*3-SMAD2/3-NEDD9 signaling pathway.

## 4. Discussion

In this study, we provided evidence that RS treatment modulates neuroprotection in the hippocampus of mice that had undergone the CUMS procedure and we identified the TGF*β*3-SMAD2/3-NEDD9 signaling pathway as a potential molecular mechanism. As RS is a water extract of the dry root of the Lamiaceae plants, *Scutellaria baicalensis* Georgi, we also detected the major components including baicalin, wogonoside, baicalein, and wogonin in the RS extract and found that the average proportions of baicalin are the highest. A large number of evidences have indicated that baicalin can protect neurons [28–31], so it is speculated that baicalin may be the main material basis, which requires further study.

So far, no animal model has been able to perfectly replicate the depressive-like phenotype currently observed in humans. It is believed that CUMS is the most commonly used, reliable, and effective rodent model of depression [32, 33]. Consistent with previous study [18], our results suggest that mice exposed to CUMS can display depressive-like behavioral deficits, including anhedonia and desperate behaviors. There is a decrease in the sucrose consumption ratio, an increase in the immobility time of FST and TST, and a decrease in spontaneous activity in CUMS-induced mice. When the treatment with RS was given for 4 weeks, the anhedonia and desperate behaviors caused by CUMS were reduced. In our previous study [34], we have shown that there was no significant alteration in locomotor activity between the control group and control treated with RS group, which revealed that RS did not cause central nervous system excitability. So we think the effect of RS in alleviating the depressive-like behaviors through its potential antidepressant-like effects.

Clinical studies have shown that patients with chronic depression have smaller hippocampal volume [35] and lower levels of cell proliferation than healthy controls [30]. The neurogenesis hypothesis explains how the hippocampal volume is decreased by the decrease in neurogenesis in the hippocampus [10]. Apoptosis is one of the prominent processes for regulating neurogenesis, including that in adult NSCs, migration neuroblasts, immature neurons, and mature neurons [36]. The decrease in the formation of new neurons in the hippocampus leads to the onset of depression, and enhanced adult hippocampal neuron formation is necessary for successful antidepressant treatment [37]. DCX has been widely used as an immature neuronal marker, and MAP2 and NeuN are the markers of neuronal maturation [38]. Previous studies have shown that CUMS could reduce DCX- and NeuN-positive cells [34, 39]. Therefore, we hypothesized that maintaining a normal hippocampal and neuronal population is critical in chronic depression therapy. In our study, CUMS resulted in decreased Nissl bodies, decreased expression of BCL-2, and increased expression of BAX. After RS treatment, the increase in neuronal apoptosis caused by CUMS was reduced. Meanwhile, the number of DCX-positive cells, NeuN-positive cells, and MAP2-positive cells in the hippocampus was decreased after CUMS, while RS administration could increase the number of positive neurons. These results indicated that RS can protect neurons by inhibiting neuronal apoptosis and increasing neuronal survival and maturation.

TGF*β*s are well known for their ability to enhance neurogenetic and neuroprotective functions [12, 14]. In the nervous system, TGF*β*3, one of the isoforms of TGF*β*s, is found in neural progenitor cells, differentiating neurons and radial glial cells, and later in mature astrocytes and numerous neuron populations [12]. The SMAD2/3 protein is a direct substrate of TGF*β*3 and can be activated to phosphorylated SMAD2/3 (p-SMAD2/3). TGF*β*3 can antagonize apoptosis after ischemia by repairing DNA damage [40], and TGF*β*3 can play a neuroprotective role through the Smad3 signaling system [41]. In the CUMS model, the expression levels of TGF*β*3 protein of the hippocampal tissues were downregulated, but electroacupuncture therapy could upregulate the TGF*β*3 protein level [13]. Neural precursor cell expressed, developmentally downregulated 9 (NEDD9) is initially found in the embryonic brain and then downregulated during development, but it remains enriched in neural precursor cells [42, 43]. NEDD9 is induced by TGF*β* and directly interacts with SMADs in various types of cells [44, 45]. Like TGF*β*, it is implicated in diverse biological processes including cell attachment, migration, and invasion as well as apoptosis and cell cycle regulation [46]. TGF*β*2/3 double-knockout mice have fewer neurons in the developing cerebral cortex and hippocampus, which is dependent on the activation of SMAD signaling and the induction of the focal adhesion protein NEDD9 [47]. The study of *Nedd9* expression and function in the nervous system found that NEDD9 is required for the maintenance of dendritic spines in the hippocampus, and NEDD9-knockout mice showed deficits both in the ability to learn the task and in their ability to recall the platform location [48]. Therefore, TGF*β*3-SMAD2/3 and NEDD9 play an important role in neuronal survival, maturation, and apoptosis.

RS is commonly used in many antidepressant Chinese medicine prescriptions [49–51]. Baicalein, a flavone derived from RS, has been reported to inhibit cancer cell metastasis via inactivation of the TGF*β*-SMAD pathway [15] and inhibit cancer cell proliferation via suppression of NEDD9 expression in cells [52]. However, the role of TGF*β*3-SMAD2/3-NEDD9 in depression and the association of RS and TGF*β*3-SMAD2/3-NEDD9 are not clear. In our study, the CUMS procedure reduced the expression of TGF*β*3, decreased the phosphorylation of SMAD2/3, and led to a decrease in NEDD9 levels. RS could reverse the CUMS-induced decrease in TGF*β*3 protein, promote the phosphorylation of SMAD2/3, and increase the expression of downstream NEDD9 protein. These results showed that RS could mediate the TGF*β*3-Smad2/3-Nedd9 signaling pathway, which might be the potential mechanism of the neuroprotective effect of RS. Importantly, it is the first time to show that the TGF*β*3-SMAD2/3-NEDD9 signaling pathway took part in the process of CUMS-induced depressive-like behaviors and the antidepressant effects of RS.

In summary, RS can improve the depressive-like behaviors through upregulating the levels of TGF*β*, p-SMAD2/3, and NEDD9 protein as well as increasing the number of DCX-, MAP2-, and NeuN-positive cells in the hippocampus. These results provide an alluring prospect that RS is used to treat depression in clinic.

## Figures and Tables

**Table 1:** Contents of four compounds in RS.

Contents^∗^ of four compounds (*n* = 3)				
Analytes^∗^	1	2	3	4
Mean (mg/g)	217.77	42.77	7.14	2.08
SD	1.96	0.38	0.10	0.02

^∗^1: baicalin, 2: wogonoside, 3: baicalein, and 4: wogonin.

**Table 2:** Calibration curve data for four reference compounds (*n* = 3).

Analytes^∗^	Regression equation	*R* ^2^	Linear range (*μ*g/ml)	LOD (*μ*g/ml)	LOQ (*μ*g/ml)
1	*Y* = 32527*X* − 17514	*R* ^2^ = 0.9993	0.84-84	0.02	0.07
2	*Y* = 37321*X* − 3879.4	*R* ^2^ = 0.9990	0.88-88	0.01	0.05
3	*Y* = 48267*X* − 137446	*R* ^2^ = 0.9991	1.81-90	0.04	0.14
4	*Y* = 56440*X* − 1223.9	*R* ^2^ = 0.9992	0.77-77	0.01	0.04

^∗^1: baicalin, 2: wogonoside, 3: baicalein, and 4: wogonin.

**Table 3:** Precision, repeatability, stability, and recovery of the analytes.

Analytes^∗^	Precision (*n* = 5)		Repeatability (*n* = 6)	Stability (*n* = 6)	Recovery (*n* = 3)	
	Intraday RSD (%)	Interday RSD (%)	RSD (%)	RSD (%)	Mean (%)	RSD (%)
1	2.91	1.12	2.64	2.13	102.8	3.70
2	1.06	1.69	3.22	1.45	95.6	3.94
3	0.90	1.57	1.73	2.50	95.7	3.72
4	0.65	1.49	2.03	1.81	95.6	2.56

^∗^1: baicalin, 2: wogonoside, 3: baicalein, and 4: wogonin.

## Data Availability

The data generated and analyzed during this study are available on request from the corresponding author.
